# Self-Adhesive and Antioxidant Poly(vinylpyrrolidone)/Alginate-Based Bilayer Films Loaded with *Malva sylvestris* Extracts as Potential Skin Dressings

**DOI:** 10.1021/acsabm.2c00254

**Published:** 2022-05-18

**Authors:** Marco Contardi, Amin Mah’d Moh’d Ayyoub, Maria Summa, Despoina Kossyvaki, Marta Fadda, Nara Liessi, Andrea Armirotti, Despina Fragouli, Rosalia Bertorelli, Athanassia Athanassiou

**Affiliations:** † Smart Materials, Istituto Italiano di Tecnologia, Via Morego 30, 16163 Genova, Italy; ‡ Dipartimento di Informatica Bioingegneria, Robotica e Ingegneria dei Sistemi (DIBRIS), Università degli studi di Genova, Via Opera Pia 13, 16145 Genova, Italy; § Translational Pharmacology, Istituto Italiano di Tecnologia, Via Morego 30, 16163 Genova, Italy; ∥ Analytical Chemistry Facility, Istituto Italiano di Tecnologia, Via Morego 30, 16163 Genova, Italy

**Keywords:** skin dressings, poly(vinylpyrrolidone), alginate, antioxidant materials, self-adhesive materials, bilayer films, Malva sylvestris

## Abstract

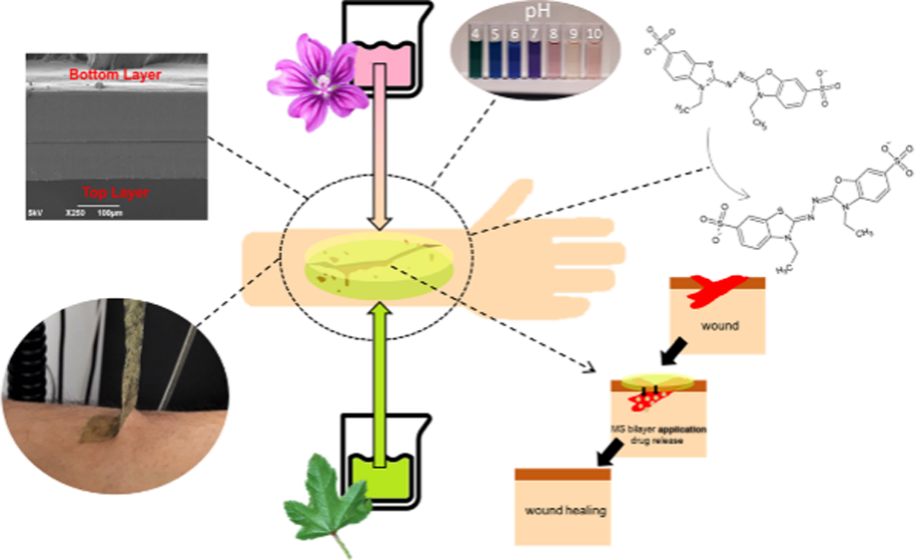

*Malva sylvestris* (MS) is a medicinal herb known worldwide for its beneficial effects due to the several active molecules present in its leaves and flowers. These compounds have shown antioxidant and anti-inflammatory properties and thus can be helpful in treatments of burns and chronic wounds, characterized mainly by high levels of free radicals and impairments of the inflammatory response. In this work, we propose bilayer films as wound dressings, based on poly(vinylpyrrolidone) (PVP) and sodium alginate loaded with *M. sylvestris* extracts from leaves and flowers and fabricated by combining solvent-casting and rod-coating methods. The top layer is produced in two different PVP/alginate ratios and loaded with the MS flowers’ extract, while the bottom layer is composed of PVP and MS leaves’ extract. The bilayers were characterized morphologically, chemically, and mechanically, while they showed superior self-adhesive properties on human skin compared to a commercial skin patch. The materials showed antioxidant activity, release of the bioactive compounds, and water uptake property. Moreover, the anthocyanin content of the flower extract provided the films with the ability to change color when immersed in buffers of different pH levels. In vitro tests using primary keratinocytes demonstrated the biocompatibility of the MS bilayer materials and their capacity to enhance the proliferation of the cells in a wound scratch model. Finally, the best performing MS bilayer sample with a PVP/alginate ratio of 70:30 was evaluated in mice models, showing suitable resorption properties and the capacity to reduce the level of inflammatory mediators in UVB-induced burns when applied to an open wound. These outcomes suggest that the fabricated bilayer films loaded with *M. sylvestris* extracts are promising formulations as active and multifunctional dressings for treating skin disorders.

## 1 Introduction

*Malva sylvestris* (commonly Mallow, here MS) is a plant that belongs to the family of Malvaceae or mallows. It grows in Europe, North Africa, and Asia and has been world widely used in folk and traditional medicine since the primitive age of humanity (ca. 3000 B.C.), with the first traces of its consumption found in primitive adult human teeth in the form of microfossils.^1^ MS shows various beneficial and therapeutic effects that have been attributed to the bioactive molecules mainly present in its leaves and flowers.^2^ Specifically, its leaves are highly rich in hydrophobic classes of compounds such as terpenoids, tocopherols, and coumarins (derivatives of hydroxycinnamic acid); sterols; and fatty acids (omega-3 and omega-6). Additionally, its flowers contain anthocyanins and anthocyanidins, a class of molecules with high antioxidant activity^3^ and capable of changing color upon pH alterations,^4^ such as malvidin, malvin, and delphinidin.^2^ Moreover, both leaves and flowers contain a wide variety of other chemical elements, such as phenol derivatives, mucilages, amino acids, proteins, and pigments.^2^ All of the aforementioned components make MS an attractive medicinal herb.

Indeed, even nowadays, the consumption of MS remains very popular among the various medicinal herbs. Its therapeutic spectrum for humans and animals^2,5^ includes anti-inflammatory, antitumor, antioxidant, antiulcerogenic,^6−9^ antiseptic, analgesic, spasmolytic, antitussive, expectorant, and diuretic^2,6,10−12^ properties. Due to the above, it has been reported to be used in traditional medicine for the treatment of diseases and pathologies. Such medicinal applications of MS can target not only various gastrointestinal disorders, genitourinary malfunctions, respiratory problems, muscular and skeletal issues, gingivitis, and tooth pains^13−15^ but also abscesses, burns, skin disorders, and injuries. Specifically for the latter, the wound healing process has been reported to be assisted by extracts from plant species, with up to 50% of traditional medicines being used for the treatment of skin conditions.^7^ Among these conditions, burns and chronic wounds are skin injuries characterized by the presence of a high level of harmful free radicals that can prolong and protract, as a chain reaction, the damage at a local and systemic level.^16^ For instance, in sunburns, the produced reactive oxygen species (ROS) can induce mutagenesis and DNA damage, triggering the release of proinflammatory cytokines such as IL-1 and IL-6.^16,17^ In chronic wounds, ROS can damage structural elements of the extracellular matrix (ECM) and cell membranes, and, together with proinflammatory cytokines, stimulate the production of serein proteinase and matrix metalloproteinases (MMPs) that can further degrade and inactivate elements of ECM and growth factors necessary to promote wound healing.^18^ Therefore, MS extracts can be beneficial for healing such injuries, with their antioxidant and anti-inflammatory activities playing a potentially crucial role in the healing procedure.

Apart from the administration of the pure extract directly onto the skin, scientists have been incorporating such extracts in the so-called “active” skin dressings to enhance the stability of the antioxidants in the formulations and ensure the proper delivery of the active molecules in the damaged skin area. Burn/wound dressings should act as a shield from the external agents, capable of absorbing the exudate produced by the skin and scavenging free radicals to accelerate the healing process and consequently limit the propagation of the damage.^19,20^ Moreover, the low cost and easy way of fabrication as well as suitable optical and mechanical properties should be taken into account for scalable production of the dressing. Freestanding films can guarantee these requirements and have been often developed as mono-, bi-, and multilayers.^21,22^

Several film materials for the treatment of burns and wounds have been designed using both synthetic and natural materials, with specific characteristics connected with their processability, cost, physicochemical properties, and interactions with tissues.^23−25^ In this work, we combined both natural and synthetic polymers in a unique material to overcome their eventual drawbacks and optimize their best features.

Poly(vinylpyrrolidone) (PVP) is a highly biocompatible, nontoxic, and chemically stable synthetic polymer, soluble in water and various organic solvents, affine to both hydrophobic and hydrophilic substances. Due to its proprieties, PVP is widely used in pharmaceutics, medicine, and cosmetics.^26,27^ Indeed, it can take various forms, something that makes it a common ingredient in drug manufacturing, for all kinds of tablets, granules, pellets, soft jelly capsules, gels, hydrogels, films and coatings, nanofiber membranes and mats, powders, syrups, oral or injectable solutions, coatings for medical devices, contact lenses, and many more.^26,28−36^

Among natural polymers, sodium alginate (Alg), a natural polysaccharide derived from brown algae, is a biodegradable, biocompatible, nontoxic, and hydrophilic biopolymer with the capacity to absorb water up to 200–300 times its weight.^37^ It has been widely utilized in the pharmaceutical field for a variety of biomedical applications, including tissue engineering, drug encapsulation, cell culture, medical tablets, and as a drug diffusion barrier.^38,39^ Furthermore, when used as a wound dressing matrix, it has the capability to release bioactive compounds, maintain a moist environment around the wound, and promote the healing of skin disorders.^40−42^

These two polymers have already been combined together for the production of hydrogels, tabs, and beads, but to the best of our knowledge, they were never combined for the fabrication of freestanding bilayer films. Moreover, Almasian and colleagues^43^ are the only ones who designed a wound dressing in the form of fibers using an MS extract, evaluating the material’s capacity to promote wound healing in diabetic mice.

Hence, in this work, we present the design and fabrication of Alg/PVP-based bilayer film materials loaded with MS leaves and flower extracts, and we explore their application as skin dressing materials. The bilayers were prepared by eco-friendly and scalable solvent-casting and rod-coating methods. The morphological, physicochemical, mechanical, and adhesive properties of MS films were thoroughly investigated. Moreover, the release profile of the extracts from the fabricated bilayers was studied. Finally, the bioactivity of MS bilayers was evaluated in both *in vitro* and *in vivo* mice models.

## 2 Materials and Methods

### 2.1 Materials

Poly(vinylpyrrolidone) (PVP; MW = 360,000), alginic acid sodium salt (Alg) with viscosity 15,000–20,000 cps, glycerol (density = 1.261 g/cm^3^), phosphate-buffered saline (PBS) solution (pH 7.4), sodium carbonate, sodium bicarbonate, sodium citrate, citric acid, potassium persulfate, 2,2′-azino-bis(3-ethylbenzothiazoline-6-sulfonic acid) diammonium salt (ABTS), and ethanol were purchased from Sigma-Aldrich and used as received. Dry MS leaves and flowers were purchased from a local pharmacy “Farmacia Svizzera” (Genoa, Italy). Deionized water was obtained from a Milli-Q Advantage A10 ultrapure water purification system.

HaCaT cells were purchased from CLS Cell Lines Service, 300493. CellTiter-Glo reagent was purchased from Promega (Madison, WI). C57BL/6J male mice were purchased from Charles River, Calco, Italy.

### 2.2 Preparation of the MS Extract

**Figure 1:**
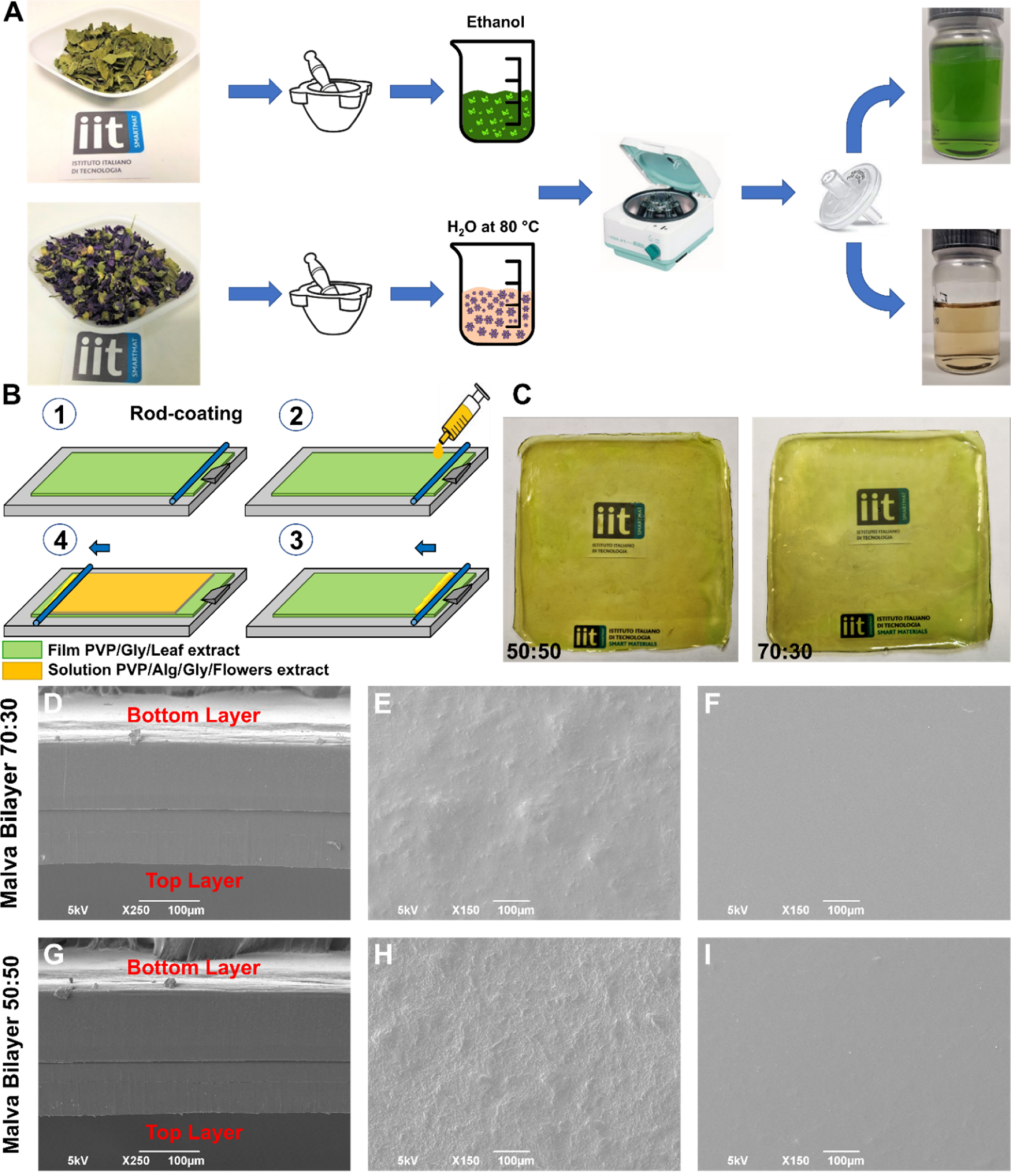
(A) Schematic representation of the preparation of the leaf and flower extracts. (B) Schematic representation of the bilayer fabricated by using the rod-coating method. Briefly, the bottom layer (in green) based on PVP and leaf extract and produced by the solvent-casting method is fixed on the rod coater (1). Then, the solution of flower extract, PVP, and Alg was injected by a syringe (2), and the rod was activated to spread the viscose solution and produce the second layer (3–4). (C) Photographs of the MS bilayer 50:50 and 70:30 samples. (D) Scanning electron microscopy (SEM) image of the cross-section of MS bilayer 70:30. (E, F) SEM images of the top view of the top layer (PVP/Alg 70:30) and bottom layer (PVP), respectively, for the MS bilayer 70:30 sample. (G) SEM image of the cross-section of the MS bilayer 50:50. (H, I) SEM images of the top view of the top layer (PVP/Alg 50:50) and bottom layer (PVP), respectively, for the MS bilayer 50:50 sample.

Initially, the dried flowers and leaves were ground separately in a marble mortar (Figure 1) to reduce their size and have better processability. The obtained flower powder was placed in a glass bottle with water at a concentration of 1.35% (w/v). The mix was heated and stirred for 2 h at 40 °C. Instead, leaf powder was mixed in ethanol at the concentration of 0.90% (w/v) and shaken for 24 h at room temperature (16–20 °C). Subsequently, both solutions were centrifuged at 5000 rpm for 30 min with a standard laboratory centrifuge. Finally, the supernatant was separated from the precipitate, and the obtained extract of flowers was filtered with a nylon filter with a pore size of 0.45 μm, while the extract of leaves was filtered with a poly(tetrafluoroethylene) (PTFE) filter with a pore size of 0.45 μm. A schematic representation of the extraction steps is reported in Figure 1A.

### 2.3 Bilayer Preparation

The bottom layer was fabricated by dissolving PVP at a concentration of 4% (w/v) and glycerol in the ethanolic solution of leaf extract. Glycerol was used as a plasticizer with a concentration of 0.5% (w/v) with respect to the total volume of the solution. The mixture was shaken for 24 h, and then, the solution was cast into plastic square Petri dishes (14 cm × 14 cm) and dried for 3 d under an aspirated hood in the dark and ambient conditions [16–20 °C and 40–50% relative humidity (RH)]. For the fabrication of the top layer, a rod coater system (model EZ coater EC-200; ChemInstruments) was used, and two solutions with different PVP/Alg weight ratios were investigated. In particular, the aqueous flower extract was mixed with glycerol at a concentration of 0.5% (w/v) and with PVP/Alg with the ratios of 70:30 (w/w) and 50:50 (w/w). A dried square bottom layer was fixed in the rod system with a snap action clip. The rod was placed at a distance of 100 μm from the surface of the dried bottom layer, and 10 mL of top layer solutions was spread close to the rod on the surface of the dried bottom layer film. Right after, the rod was activated, spreading the top layer solution from the end to the final part of the dried bottom layer film. The obtained bilayers were dried for 24 h under an aspirated hood in the dark and ambient conditions [16–20 °C and 40–50% relative humidity (RH)], resulting in final bilayer films with an average thickness of 260 ± 50 μm. A schematic representation of the preparation steps is reported in Figure 1B.

Control monolayer film materials without any extract were also produced. In particular, films of pristine PVP and PVP and Gly were prepared in ethanol, while pure Alg, Alg and Gly, PVP with Alg and Gly with a polymeric weight ratio of 70:30, and PVP with Alg and Gly with a polymeric weight ratio of 50:50 were obtained from an aqueous solution. All control films were fabricated starting from a total polymeric concentration of 4% (w/v) with respect to the used solvent.

### 2.4 Morphological Analysis

The morphology of the bilayer films was analyzed by scanning electron microscopy (SEM). The cross sections of the MS bilayers were obtained by using a razor blade. Samples for the cross section and the top view were covered with a thin layer of gold (10 nm) using a sputter coater. SEM imaging was performed using a SEM JEOL-JSM 6490, operating at an acceleration voltage of 5 kV.

### 2.5 Attenuated Total Reflection-Fourier Transform Infrared (ATR-FTIR) Spectroscopy

Infrared spectra were recorded with an ATR accessory (MIRacle ATR, PIKE Technologies) with a diamond crystal coupled to a Fourier transform infrared (FTIR) spectrometer (Vertex 70v FTIR, Bruker). All spectra were recorded from 4000 to 600 cm^–1^ with a resolution of 4 cm^–1^, accumulating 128 scans.

### 2.6 UV–Vis Spectroscopy

A Cary 300 UV–Vis spectrophotometer was used to analyze the extracts of MS. Quartz cuvettes were used for the recording of the spectra. The reference solutions in spectrophotometric measurements were pure ethanol for the leaf extract, while different aqueous solutions were utilized for the flower extract: in detail, citrate buffers for pH levels of 4 and 5, phosphate buffers to obtain solutions of pH levels of 6 and 7.4, and carbonate buffers for the pH levels of 9 and 10. The pH values were verified by a pH meter.

### 2.7 Ultraperformance Liquid Chromatography (UPLC)

All chemicals and reagents used for sample preparation and liquid chromatography–mass spectrometry (LC–MS)/MS analysis were purchased from Aldrich (Milano, Italy). All analyses were carried out using an ACQUITY UPLC system coupled to a Synapt G2 QToF high-resolution mass spectrometer (Waters, Milford, MA).

The samples were simply diluted 10× in acetonitrile/water (50:50). About 5 μL of these samples were then injected into the Acquity UPLC liquid chromatography system coupled with the Synapt G2 QToF high-resolution mass spectrometer (both from Waters, Milford, MA). The analytes were then separated on a T3 (2.1 × 100 mm) reversed-phase column (Waters) using a linear gradient of acetonitrile in water (1–100%). The eluting compounds were analyzed by high-resolution mass spectrometry in both positive and negative ion electrospray modes. The Leucine Enkephalin reference standard was used as lock-mass to achieve mass accuracy below 5 ppm. Metabolites were tentatively identified by interrogating the publicly available HMDB (Human Metabolome Database), METLIN, and LipidMaps reference databases.^44−46^

### 2.8 Mechanical and Adhesion Properties

The mechanical proprieties of the MS bilayers and control films were determined by uniaxial tension tests using a dual-column universal testing machine (Instron 3365). Materials were cut in dog bone specimens (at least 5 of them for each sample) with a width of 4 mm and an adequate length of 25 mm. Displacement was applied at a rate of 5 mm/min. The Young modulus (YM), stress at maximum load, and elongation at maximum load were extracted. All of the stress–strain curves were recorded at 18–22 °C and 55% RH.

Peel adhesion tests were carried out on the dual-column universal testing machine (Instron 3365) equipped with a custom setup on an ISO 8510 standard: a volunteer’s human arm was placed on the horizontal moving table, the MS bilayer samples were gently applied to slightly wet skin to simulate the local humidity of a wound/burn, and one end was clamped. The upper clamp was displaced upward with a constant rate of 50 mm/min; the table was moved horizontally by a pulley so that the peeling angle was stable (90°). The MS bilayer samples were placed with the bottom layer made of PVP on direct contact with the skin due to the adhesive capacity of the PVP. The skin was previously wet with 200 μL of water.

### 2.9 Drug Release

The release of the MS extracts from the bilayer material was measured by UPLC. MS bilayers 70:30 and 50:50 were placed in separate wells of a multiwell, each one containing 2.5 mL of PBS at room temperature. At specific time points, 0.5 mL of each well was collected for UPLC analysis and replaced with 0.5 mL of fresh medium. Before each measurement, the liquid in the wells was gently mixed to have good dispersion of the released drug in it. The experiments were carried out in triplicate and repeated three times. Results were expressed as a cumulative percentage.

### 2.10 ABTS Free Radical Cation Scavenging Assay

The ABTS radical cation was produced by the reaction between 2.45 mM potassium persulfate solution with 7 mM ABTS water solution in the dark at room temperature for 16 h.^47^ The ABTS solution was diluted with water until an absorbance of 1.20 au at 730 nm was obtained. After that, different amounts of flower extract were added to 3 mL of the diluted ABTS solution. The decrease in absorbance was followed by a Cary 300 Scan UV–visible spectrophotometer at 730 nm at different time points. All measurements were performed in triplicates. Radical scavenging activity was expressed as the inhibition percentage of free radical by the sample and calculated from eq 1
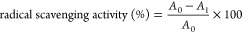
1where *A*_0_ is the absorbance value of the control (3 mL of 0.2 mM ABTS solution in water) and *A*_1_ is the absorbance value of the sample at different time points.

### 2.11 Water Contact Angle (WCA)

Static water contact angles (WCAs) of the bottom and top layer of both types of MS bilayers (70:30 and 50:50) were measured by a contact angle goniometer (OCA-20 DataPhysics, Germany) at room temperature. Deionized water droplets of 5 μL were deposited on the surface of the samples, and the contact angle was calculated from the side view with the help of built-in software. To ensure the repeatability of the result, six measurements for each sample were performed.

### 2.12 Humidity Uptake

The humidity uptake capacity of the samples was evaluated as described in Contardi et al.^48^ Dry samples were weighed (∼25 mg) and placed in different chambers with controlled humidity (RH 0, 11, 44, 84, 100%). The samples were kept in different humidity chambers until equilibrium conditions (usually 24 h), each film was then weighed, and the amount of adsorbed water moisture was estimated based on the difference between the weight of each film and its initial dry weight.

### 2.13 Biocompatibility

The *in vitro* cytotoxicity assessment was conducted on immortalized human keratinocytes—HaCaT—using the CellTiter-Glo Luminescent viability assay (Promega, MI, Italy) measuring adenosine triphosphate (ATP) levels. Cells were grown in specific culture media—Dulbecco’s modified Eagle’s medium (DMEM) high-glucose completed with 10% fetal bovine serum (FBS) and 2 mmol L^–1^l-glutamine—in a humidified incubator at 37 °C with 5% CO_2_. For cytotoxicity experiments, HaCaT cells were seeded in 96-well plates at a density of 3.5 × 10^5^ in a final-medium well volume of 100 μL and incubated until the proper confluence was reached. After 24 h of treatment, cells were rapidly rinsed with prewarmed PBS with Ca^2+^/Mg^2+^, and the cell medium was replaced with the extraction one (control samples were treated with media processed as the extraction ones), and cells were incubated for an additional time of 24 and 48 h. Extracts were prepared by placing bilayer films at different concentrations in the cell medium. Cell viability was determined by measuring ATP levels by the CellTiter-Glo assay, as indicated by the supplier as the percentage of survived cells relative to control cells. Data represent the mean ± standard deviation (SD) of three independent experiments. The impact of MS bilayer films on cell morphology was also monitored using a LEICA DMI6000B inverted microscope.

### 2.14 Wound Scratch

As previously described by Contardi et al.,^49^ cells were seeded into 24-well plates at 30 × 10^4^ cells and maintained at 37 °C and 5% CO_2_ for 24 h to permit cell adhesion and the formation of a confluent monolayer. At this stage, cells were wounded with a sterile plastic pipette tip to create a scratch with an approximate width of 0.4 mm. The cells were then washed twice with PBS, and the medium was replaced with MS bilayer and control PVP + Gly + Alg 70:30 and 50:50 film extracts. All scratch assays were performed in triplicate. The wound closure was monitored, collecting digitized images immediately after scratching, 24 and 48 h postinduction. Images were analyzed by ImageJ software (NIH). Data were reported as the extent of wound closure calculated on the initial scratch width.

### 2.15 *In Vivo* Analysis

#### 2.15.1 Animals

Eight-week-old C57BL/6J male mice were used (Charles River, Calco, Italy) for animal models. Animals were grouped in ventilated cages and had access to regular food and water. They were maintained under controlled conditions: temperature (21 ± 2 °C), humidity (50 ± 10%), and light (12/12 h of light/dark, respectively). All animal experiments were performed according to the guidelines established by the European Communities Council Directive (Directive 2010/63/EU of 22 September 2010) and approved by the National Council on Animal Care of the Italian Ministry of Health. All efforts were made to minimize animal suffering and use the lowest possible number of animals required to produce statistically relevant results, according to the “3Rs concept”. Animals were anesthetized with a mixture of ketamine (10%) and xylazine (5%), and dorsal skin was shaved with an electric clipper. Mice were housed individually during the experiments, with food and water *ad libitum*.

One full-thickness skin wound was produced at the center of the back of each animal, utilizing a sterile 6 mm biopsy punch. The wounds were photographed and then covered with the MS bilayer 70:30 films (*n* = 5 for each experimental group). To avoid the removal of the dressings when the mice were awake, all treated wounds were covered with Tegaderm just before the mice woke up, until the end of the experiment. Photographs were taken at different time intervals (1, 5, 15, 30, 60, 120, 240, 360 min, and 24 h) after the dressing application to evaluate bioresorption over time. ImageJ software was used to analyze the photographs and calculate the bioresorbed membrane percentage during the time.^30^

Burn wounds were induced, as reported in Hajiali et al.^41^ Briefly, mice were placed in a tube of UVB opaque material with a squared opening of approximately 1.5 cm^2^ in the desired portion of skin and exposed to a narrowband UVB light source (TL01 fluorescent tubes, Philips, U.K.), λ_max_ = 312 nm (maximal dose of 1000 mJ/cm^2^). Immediately after burn induction, the exposed area was treated by placing MS bilayer films and covering it with Tegaderm to prevent the removal of the treatment by the mice. Nonirradiated mice followed the same procedure without being UVB-exposed (*n* = 5 animals per experimental group). Animals were sacrificed 48 h post-UVB burn induction, and samples from UVB-exposed and nonexposed mice skin were taken and stored at −80 °C until processing.

#### 2.15.2 ELISA Assay

Skin samples from naïve, Sham, and bilayer film-treated mice were collected after 48 h postirradiation induction. Each sample was homogenized and subsequently centrifuged, and the supernatant was isolated and stored at −80 °C. Cytokine (IL-6 and IL-1β) expression was measured using the ELISA Quantikine kit (R&D Systems), according to the manufacturer’s instructions.

### 2.16 Statistical Analysis

For the wound scratch assay, analysis of variance (ANOVA) was utilized to investigate the statistical significance, followed by Bonferroni’s posthoc test. GraphPad Prism 5 was utilized for all statistical analyses (GraphPad Software Inc., San Diego, CA). Outcomes with *p* values <0.05 were considered statistically significant.

For the investigation of the IL-6 and IL-1β levels in *ex vivo* tests, ANOVA was used to evaluate statistical significance, followed by Tukey’s posthoc test. GraphPad Prism 5 was used for all statistical analyses (GraphPad Software Inc., San Diego, CA). *P* values < 0.05 were considered significant.

## 3 Results and Discussion

### 3.1 Morphological Analysis

Bilayer polymeric materials were designed to ensure the encapsulation of bioactive molecules from both the components of MS (flowers and leaves). Bioactive molecules from flowers were extracted by hot water due to the excellent solubility of anthocyanins—mainly present in this part of the plant—in water. Instead, for the leaves, ethanol was selected as a solvent for the extraction of their bioactive molecules, such as hydroxycinnamic acid derivatives, which are highly soluble in an alcoholic solvent. The aqueous flower extract was added to the top layer. This is because the Alg, present in this layer, is not soluble in ethanol; therefore, it was mixed with the aqueous extract. On the contrary, PVP is soluble in both solvents, and the ethanolic leaf extract was used to fabricate the bottom layer, which is entirely composed of PVP. Therefore, the MS bilayers are designed to be applied with the PVP side in direct contact with the skin (skin side), also because of the adhesive properties of PVP, and the PVP/Alg-based layer loaded with the flower extract on the external side (air side).

**Figure 2:**
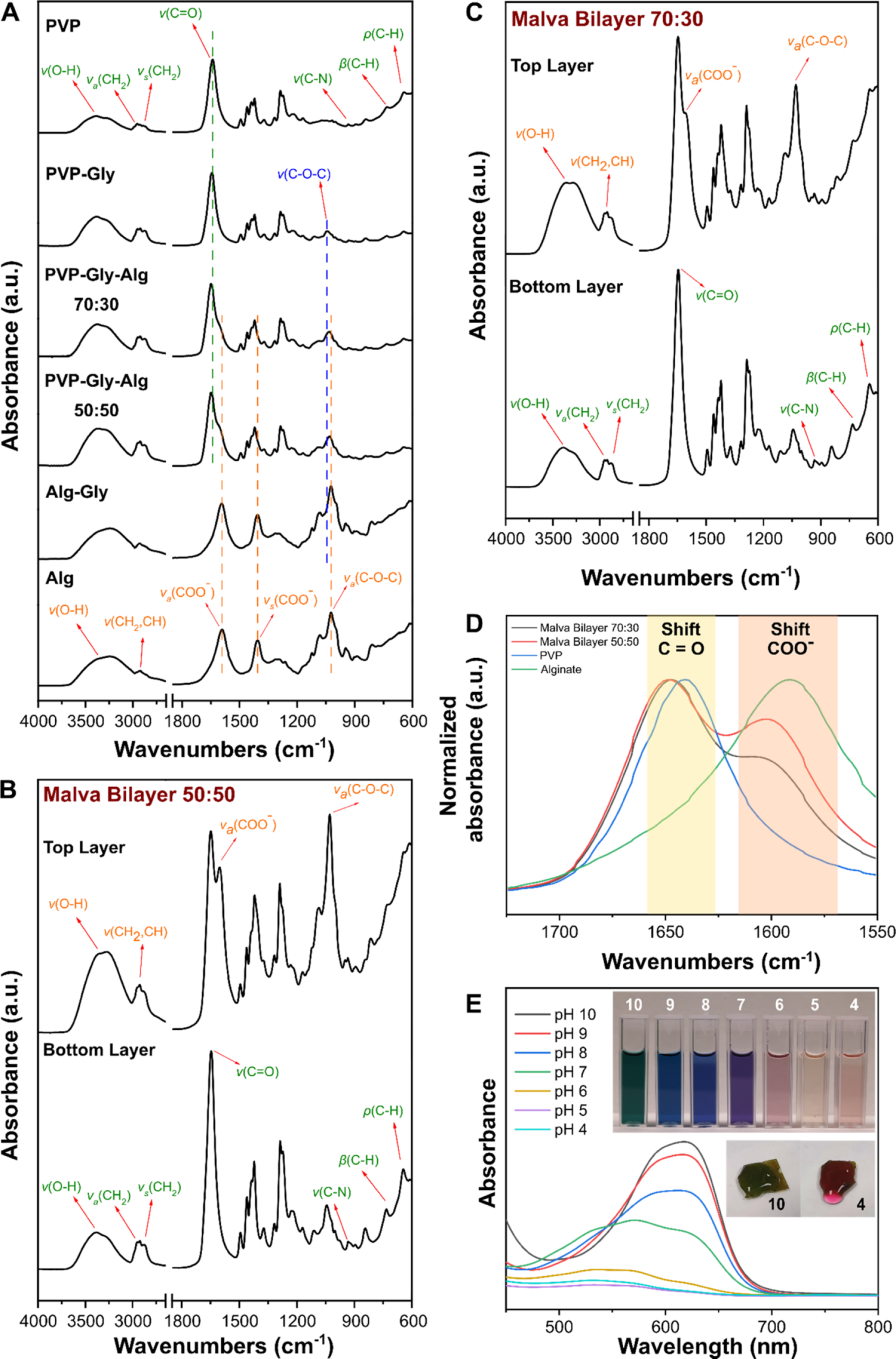
(A) ATR-FTIR spectra of PVP, PVP–Gly, PVP–Gly–Alg 70:30, PVP–Gly–Alg 50:50, Alg–Gly, and Alg samples. (B) ATR-FTIR spectra of the top and bottom layer of the MS bilayer 50:50 sample. (C) ATR-FTIR spectra of the top and bottom layers of the MS bilayer 70:30 sample. (D) Comparison in the spectral region of the C=O and COO^–^ stretching modes (1750–1550 cm^–1^) of PVP, Alg, and MS bilayer 50:50 and 70:30 samples. (E) UV–vis spectra of flower extract at different pH levels. The insets show the flower extract at pH from 10 (left) to 4 (right) and the MS bilayer 70:30 in contact with drops of aqueous solutions at pH 10 (left) and 4 (right).

Photographs of MS bilayers 50:50 (left) or 70:30 (right) PVP/Alg are reported in Figure 2C. The resulting films have a homogeneous green color and transparency. The morphology of the bilayer was investigated by SEM. Figure 1D shows the cross-sections of the MS bilayers 50:50 and 70:30, and a well-defined separation of the two layers can be noticed in both samples. However, by analyzing the surface of the top layer, morphological differences could be observed. Indeed, in the top layer of MS bilayer 70:30, a rough surface was noted (Figure 1E), and it was even more evident when the ratio between PVP and Alg became 50:50 (Figure 1H). On the other hand, the PVP-based surface of the bottom layer in both MS bilayer samples was fully smooth.

### 3.2 Chemical Characterization of the Films and Extracts

The monolayer and bilayer films were chemically characterized by ATR-FTIR spectroscopy. The ATR-FTIR spectra of the control monolayer films are reported in Figure 2A. PVP typical vibration modes were found:^50^ O–H stretching mode at 3403 cm^–1^; asymmetric and symmetric CH_2_ stretching modes at 2988, 2955, 2924, and 2864 cm^–1^; C=O stretching mode at 1641 cm^–1^; C–N stretching mode at 1018 cm^–1^; out-of-plane C–H bending mode at 844 cm^–1^; and C–H rocking mode at 733 cm^–1^. In the PVP–Gly sample, the C–O–C stretching mode of the glycerol appears at 1043 cm^–1^. In the Alg sample, the typical IR vibration modes were observed: O–H stretching modes at 3383 and 3231 cm^–1^; asymmetric and symmetric CH_2_ and CH stretching modes at 2961, 2936, 2918, 2874, and 2849 cm^–1^; asymmetric and symmetric COO^–^ stretching modes at 1591 and 1406 cm^–1^; and symmetric C–O–C stretching mode at 1024 cm^–1^. In Alg–Gly, a tiny shoulder at 1043 cm^–1^ attributed to the C–O–C stretching mode of Gly was found. The spectra of PVP–Gly-Alg 50:50 and 70:30 samples showed overlapped vibration modes of the three components. The ATR-FTIR spectra of MS bilayers 50:50 and 70:30 are shown in Figure 2B,C, respectively. For both samples, the spectra of the top and bottom layers are reported. In both cases, in the top layer, the peaks of PVP and Alg could be noticed, while in the bottom layer, mostly PVP vibrations modes were observed. No peaks directly connected with the extract were found. These results suggested a well-defined separation of the composition in the two layers after the fabrication process.

However, in the MS bilayer samples, shifts in the C=O stretching mode of PVP and in the COO^–^ stretching mode of Alg were observed, as highlighted in Figure 2D. In detail, the C=O peak shifted from 1641 to 1649 cm^–1^, compared to the pristine PVP with the MS bilayer samples. Similarly, the COO^–^ peak of Alg moved from 1591 to 1603 cm^–1^. The presence of the extract inside the polymer matrix, even if not directly detectable by the observation of new peaks in the spectra, could be the reason why the shifts in C=O and COO^–^ groups of the two polymers were observed, probably due to the establishment of new interactions or due to their involvement in H-bonds with the polar groups of the molecules of the extracts.

The two extracts were characterized by UV–vis spectroscopy. The leaf extract in the UV–vis spectrum presents the typical absorption wavelengths of chlorophyll.^51^ UV–vis spectra of the flower extract at different pH levels were also acquired to confirm the presence and verify that the extraction process did not affect the capacity of anthocyanins to change color (Figure 2E). As it can be noticed, the flower extract changed its spectrum profile as a function of the pH, and its color became green/blue at high pH and pink/red at low pH. This feature is typical of anthocyanins/anthocyanidins and suggests that the chemical structure of the molecules was not affected. A similar effect was also presented in the MS bilayer films when they were in contact with drops of solutions with different pH levels (10 and 4), as shown in the inset in Figure 2E. The color change property can help detect variations in the physiological pH in a wound. In fact, different conditions can make the pH change in a wound bed environment, usually in the range from 10 to 4, as a function of the occurring alteration (for instance, prolonged inflammation, bacterial strains).^52−54^

The leaf and flower extracts were characterized by UPLC. The chromatograms are shown in Figures S2 and S3. Both extracts were rich in active molecules, and the lists of the found molecules are reported in Table S1. Anthocyanins such as malvidin were only present in the flower extract, while the leaf extract was rich in ferulic acid. For this reason, these two molecules were chosen as markers for the two different layers and were followed during the drug release experiments.

### 3.3 Mechanical and Adhesive Properties

**Figure 3:**
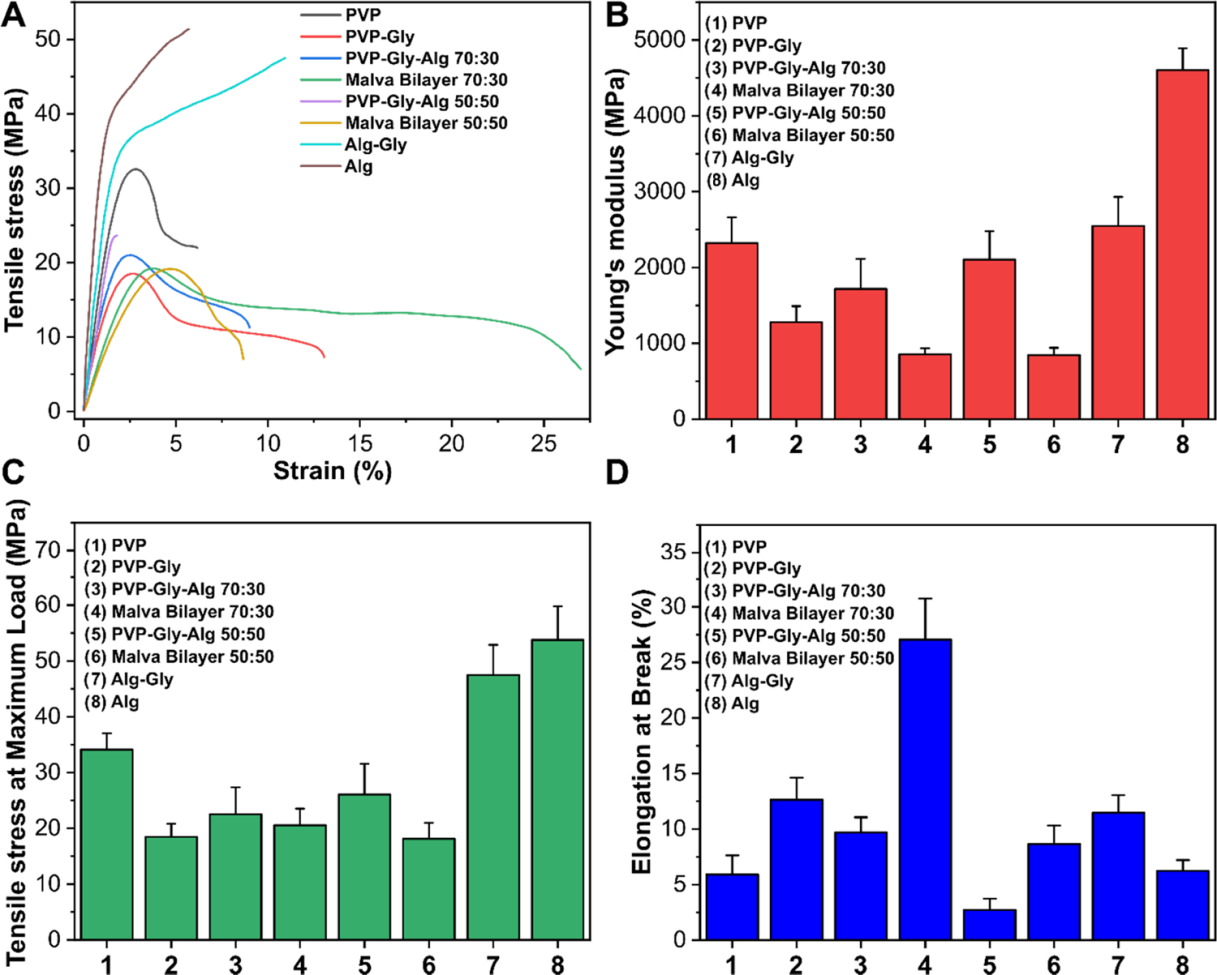
(A) Stress–strain curves of PVP and alginate-based films. (B–D) Values of Young’s modulus, tensile stress at maximum load, and elongation at break of the PVP and alginate-based films, respectively.

The mechanical properties of the MS bilayers and control samples were investigated. Typical stress–strain curves for all of the samples are reported in Figure 3A. Instead, the values of Young’s modulus (YM), tensile stress at maximum load (TS), and elongation at break (EAB) are shown in Figure 3B,C,D. Pristine PVP showed the typical profile of a rigid and fragile material, with a YM of ≈ 2300 MPa, a TS of ≈ 34 MPa, and an EAB of ≈5.9%. Also, films of pure Alg had an elevated value of YM ≈ 2500 MPa, TS of 47 MPa, and EML of ≈6.3%. In both cases, the introduction of Gly in the polymeric structure induced a plasticizing effect with the reduction of YM and TS values and an increase of the EAB. Instead, when PVP and Alg were combined in the weight ratios of 70:30 and 50:50, a slight reduction of the YM and TS with respect to the PVP and Alg samples was noticed. Finally, the bilayer samples 70:30 and 50:50 loaded with MS extracts showed a decrease of YM values to ≈850 MPa for both the samples, while the TS values were 20 and 18 MPa, respectively. Furthermore, as can be noticed in Figure 3A, MS bilayer 70:30 displayed the highest value of elongation at break of ≈27% among the analyzed samples. This enhancement of the mechanical properties of the MS bilayer samples with respect to the control samples can be explained by the presence in the extracts of molecules with exposed OH groups, such as anthocyanins and ferulic acid, which can interact with the polymeric chains and change their mechanical behavior.^34^

On the other hand, for the samples with PVP/Alg 50:50, a general reduction of the elongation was noticed with respect to the samples with PVP/Alg 70:30. This behavior can be explained because a stronger affinity of the polymeric chains in establishing H-bonding interactions among the two polymers instead of the small molecules present in the extract can occur.^55^ All of the found values for the developed samples are reported in Table 1.

**Table 1:** Values of Young’s Modulus, Tensile Stress at Maximum Load, and Elongation at Break for All of the Samples under Investigation^a^

samples	Young’s modulus (MPa)	tensile stress at maximum load (MPa)	elongation at break (%)
PVP	2323 ± 339	34.1 ± 2.9	5.91 ± 1.72
PVP–Gly	1279 ± 210	18.5 ± 2.3	12.64 ± 2.00
PVP-Alg–Gly 70:30	1716 ± 397	22.5 ± 4.8	9.70 ± 1.36
MS bilayer 70:30	853 ± 80	20.5 ± 3	27.05 ± 3.70
PVP-Alg–Gly 50:50	2105 ± 376	26 ± 5.5	2.71 ± 1.01
MS bilayer 50:50	847 ± 95	18.1 ± 2.9	8.65 ± 1.66
Alg–Gly	2547 ± 383	47.5 ± 5.3	11.48 ± 1.58
Alg	4602 ± 291	53.8 ± 6	6.23 ± 0.97

a The results are expressed as average ± standard deviation (*n* ≥ 7).

**Figure 4:**
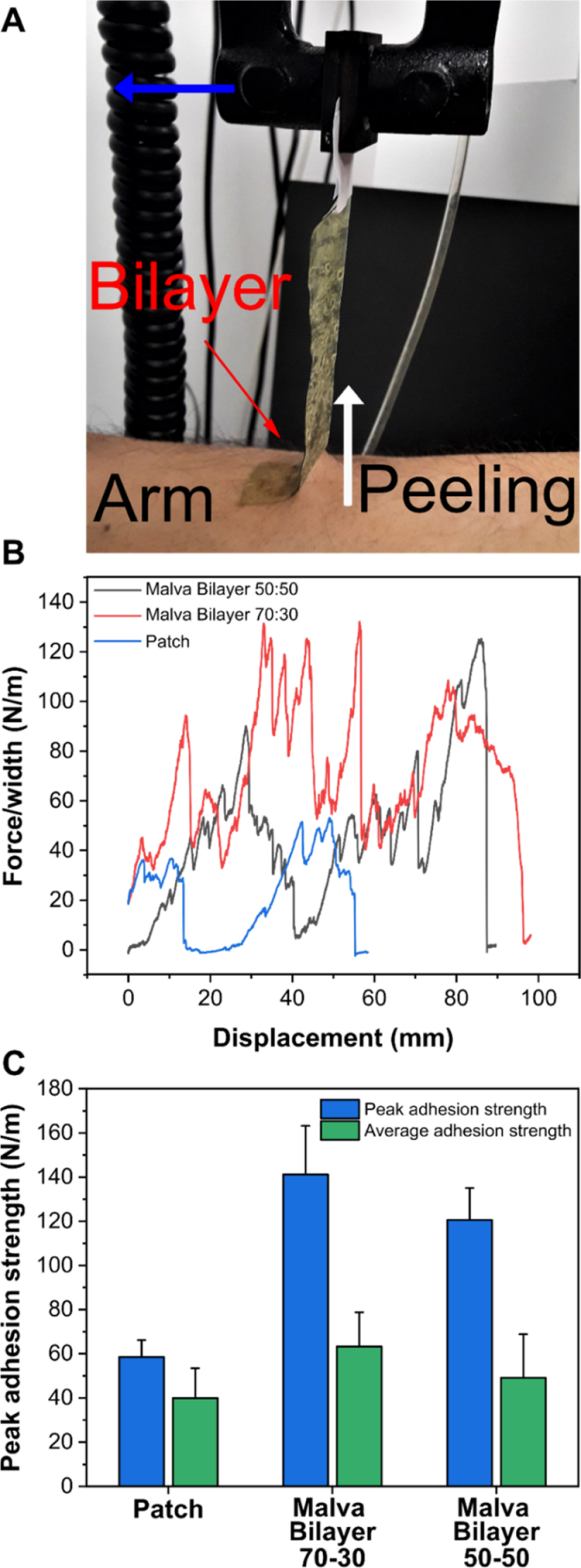
(A) Photograph obtained during the 90° peel test; the arrows indicate the displacement direction. (B) Peel adhesion measurements (on a human arm) for the commercial patch adhesion (blue line) and the MS bilayer 50:50 (gray line) and 70:30 (red line) films. (C) Histogram of the peak and average adhesion strength obtained during the 90° peeling test for the commercial patch and MS bilayers 50:50 and 70:30.

Furthermore, the adhesive properties of MS bilayer 70:30 and 50:50 samples to the human skin were investigated. Peeling tests were performed for both samples, and the results were compared to a commercial patch. As shown in Figure 4A, the patches were placed with the PVP bottom layer on the slightly wet skin and pulled at an angle of 90° with respect to the human skin. Typical peel force versus displacement profiles are reported in Figure 4B. As can be noticed, both the MS bilayer samples, 70:30 and 50:50, had strong self-adhesive properties to the skin, with average adhesion strengths of 63 and 49 N/m and with peaks of adhesion of 141 and 120 N/m, respectively. These results demonstrated a superior adhesion to the skin in comparison not only to the commercial patch, see Figure 4C, but also to other patches and PVP-based films previously produced and reported in the literature.^30,56−59^ This effect can be justified by the presence of Gly and extract molecules that can increase the interaction points between the materials and the human skin.^60^

Therefore, the MS bilayer samples are suitable for application to the skin due to their ductile and adhesive behavior. These skills can allow the application of these patches in different parts of the body and resist the mechanical force/stress induced by the patients’ movements or external forces.

### 3.4 Release, Scavenging, WCA, and Water Uptake Properties

**Figure 5:**
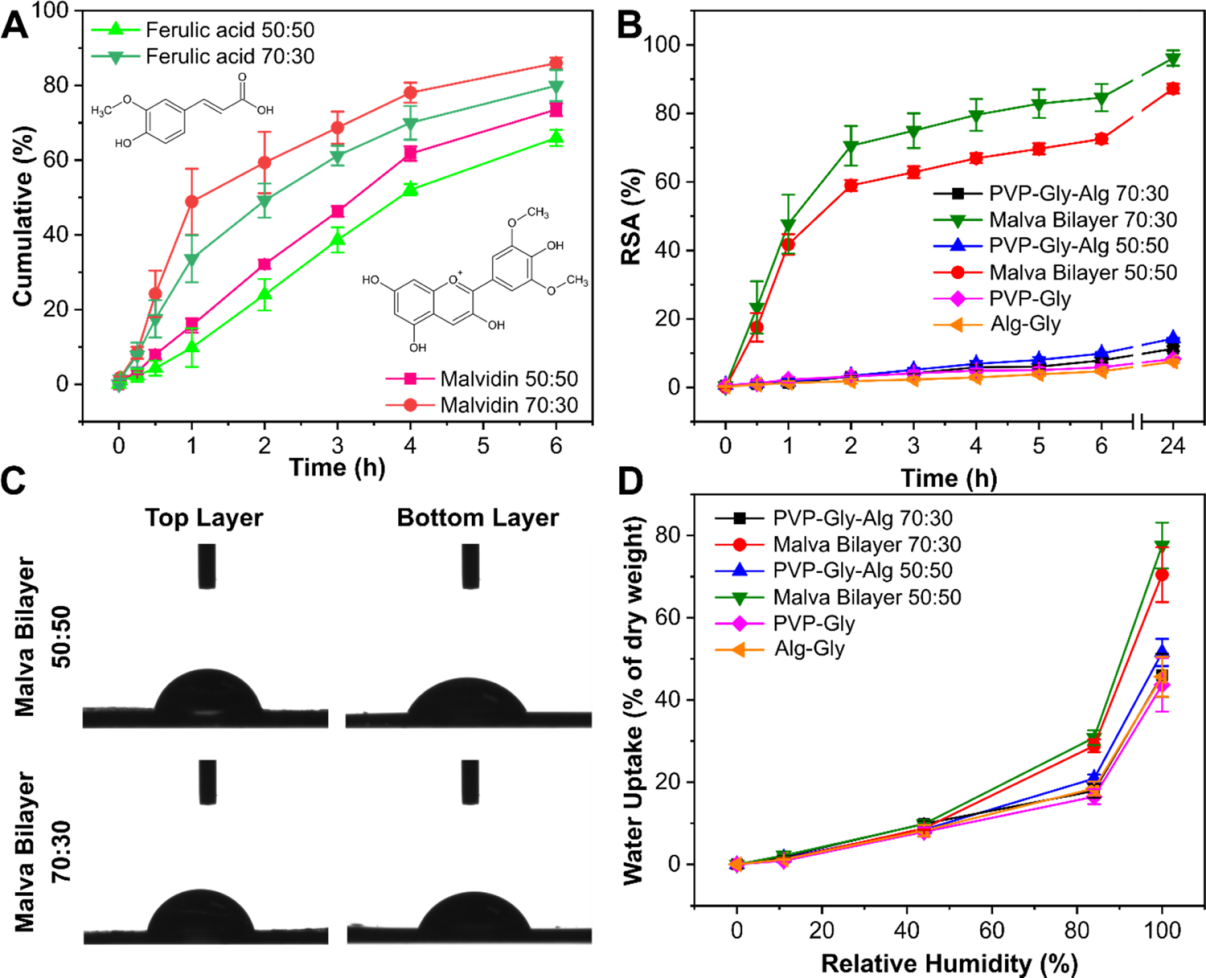
(A) Release profile of malvidin and ferulic acid from the MS bilayer films over a period of 6 h. (B) Percentage of scavenging activity for the MS bilayers and the control films. (C) Results of water uptake analysis for the MS bilayers and the control films. (D) Photographs of the WCA measurements of the top and bottom layer for the MS bilayers.

The release in PBS medium from MS bilayers 50:50 and 70:30 of the two bioactive compounds was evaluated, and the results are reported in Figures 5A and S4. As mentioned before, malvidin was used as a marker for the release from the top layer (flowers extract) in both bilayers, while ferulic acid was the marker for the bottom layer (leaf extract). The overall release was followed for 96 h, and the release profiles are reported in Figure S4. The main differences could be observed in the first 6 h. Indeed, malvidin released from MS bilayer 50:50 was 73%, while that from bilayer 70:30 was 85% (Figure 5A). Similarly, the diffusion of ferulic acid was slightly faster in sample 70:30 with respect to that in the 50:50 one, releasing 80 and 66% of the total loaded amount in the first 6 h, respectively. This effect can be justified by the composition of the top layer, where different quantities of the Alg are present, which is less soluble in water with respect to PVP. The slight difference in the initial diffusion between malvidin and ferulic acid from both the MS bilayers can be justified by the different solubility of the two components in water. This fast release profile is suitable for the treatment of both fresh burns and wounds since the material can quickly scavenge the free radicals that can expand the damage to the surrounding tissue.^61^

The antioxidant properties of the MS bilayers and the control samples were investigated by the ABTS assay, and the main results are reported in Figure 5B. As can be seen, none of the control films showed a significant antioxidant response. Instead, MS bilayers 70:30 and 50:50 showed radical scavenging activities of 87 and 96%, respectively. These outcomes demonstrate a strong antioxidant activity of the bilayer materials attributed to the presence of the MS extracts.

WCA was evaluated to characterize the surface properties of both of the bilayers’ faces. Photographs of the WCA measurements are reported in Figure 5C. For the top layer of MS bilayer 50:50, the WCA was 82.6 ± 0.5°, while the bottom face showed a WCA of 69.8 ± 3.7°. Similarly, in MS bilayer 70:30, the top layer presented a slightly higher WCA of 83.2 ± 1.5°, while the bottom face had a WCA of 73.5 ± 3.0°. This difference in the wettability properties of the two faces can be a consequence of the different surface morphology found between the two layers in the MS bilayer samples (Figure 1E,F,H,I).

Both wounds and burns can release exudate, which can be full of harmful free radicals that can locally propagate the damage. For this reason, it is essential that the burn/wound dressings absorb the produced exudate and release the antioxidants loaded in the polymeric matrices. Figure 5D displays the moisture uptake results for the MS bilayers and the control samples. All of the pristine PVP and Alg-based films were able to absorb approx. 40–50% of moisture with respect to their original weight. Instead, MS bilayers 70:30 and 50:50 showed a superior capacity of moisture absorption of approx. 70–80%. This can be justified by the compounds present in the extracts that can enhance the ability of the material to absorb water.^62^

The capacity of MS bilayers to absorb water from humidity and in wet conditions is a significant cause of their adhesive properties. At the same time, the polymeric matrices can absorb the moisture of the skin, conferring a stable adhesion to the tissue.

### 3.5 *In Vitro* Biocompatibility, Proliferation, and Anti-Inflammatory Properties

**Figure 6:**
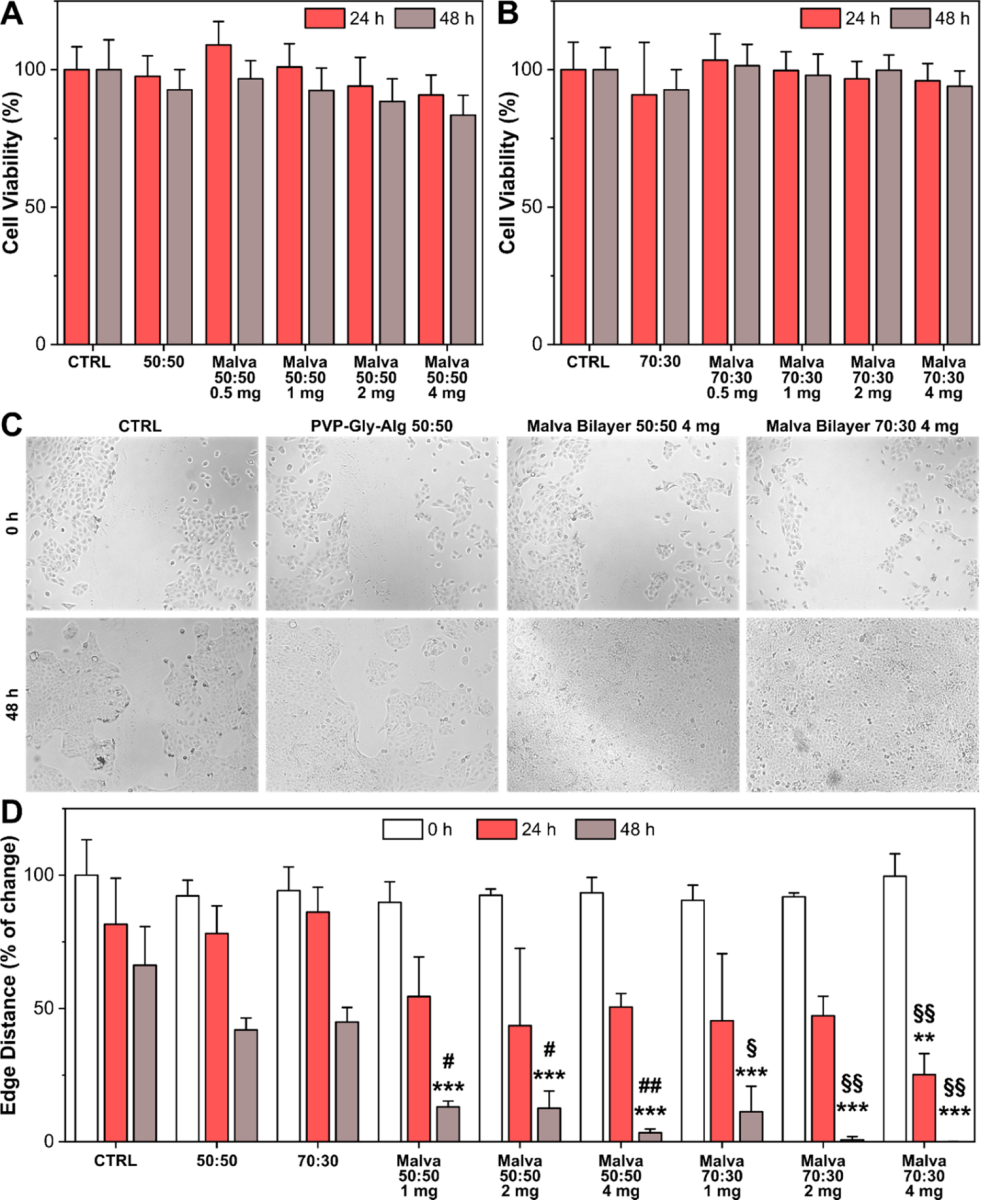
(A) Histogram of cell viability of HaCaT cells incubated for 24 and 48 h with MS bilayer 50:50. PVP–Gly-Alg 50:50 was also evaluated as a control of the polymeric system. (B) Histogram of cell viability of HaCaT cells incubated for 24 and 48 h with MS bilayer 70:30. PVP–Gly-Alg 70:30 was also evaluated as a control of the polymeric system. (C) Images of nontreated HaCaT cells (CTRL) and cells treated with 4 mg/mL PVP–Gly-Alg 50:50, MS bilayer 50:50, and MS bilayer 70:30, immediately after and 48 h after the creation of the wound scratch. (D) Quantification of the edge distance of the wound scratch at 0, 24, and 48 h. Results are presented as the average ± SD. **p < 0.01 vs CTRL; ***p < 0.001 vs CTRL; ^#^*p* < 0.05 vs 50:50; ^##^*p* < 0.01 vs 50:50; ^§^*p* < 0.05 vs 70:30; ^§§^*p* < 0.01 vs 70:30.

The biocompatibility of MS bilayers 70:30 and 50:50 was tested on the HaCaT cell line by the CellTiter-Glo assay. PVP–Gly-Alg 70:30 and 50:50 without the MS extracts were also tested as controls to evaluate any side effects of the MS extract or efficacy due to the polymeric systems. The cell viability results of different concentrations of MS bilayers 70:30 and 50:50, from 0.5 to 4.0 mg/mL, and 4 mg of PVP–Gly-Alg 70:30 and 50:50 after 24 and 48 h are reported in Figure 6A,B, respectively. According to ISO10993-5 guidelines, as the cell viability of the sample extracts was higher than 70% of the control group, all materials were considered biocompatible. In addition to this, the capacity of the biomaterials to affect the proliferation of keratinocytes was evaluated using a wound scratch model. Photographs and the main results of the wound scratch experiments are reported in Figure 6C,D, respectively. The edge distance of the wound was evaluated at 0, 24, and 48 h, and MS bilayers were tested at concentrations from 1 to 4 mg/mL, while the polymeric controls were tested at a concentration of 4 mg/mL. A slight effect of the polymeric systems with respect to the nontreated cells can be observed, something that can be justified by the presence of Alg and its well-known proliferative effects on cell models. Instead, MS bilayers at all tested concentrations showed a statistical improvement in the wound healing after 48 h, both with respect to the CTRL and to the corresponding polymeric control samples. Furthermore, at a concentration of 4 mg/mL, MS bilayer 70:30 significantly ameliorated the healing of the scratch already from the first 24 h even compared to MS bilayer 50:50. This outcome can be justified by the differences in the release of bioactive molecules between the two bilayers. Indeed, as already proved, MS bilayer 70:30 showed a slightly faster release of malvidin and ferulic acid compared to MS bilayer 50:50.

### 3.6 Bioresorption and Anti-Inflammatory Properties in *In Vivo* Mice Models

For the *in vivo* investigations, MS bilayer 70:30 was selected and used due to its slightly better performance in the wound antioxidant and wound scratch tests, probably connected with a faster release of the bioactive molecules present in the MS extract. Moreover, the 3R guidelines suggest minimizing the total number of animals used to obtain reliable statistical results. Thus, we focused on MS bilayer 70:30.

**Figure 7:**
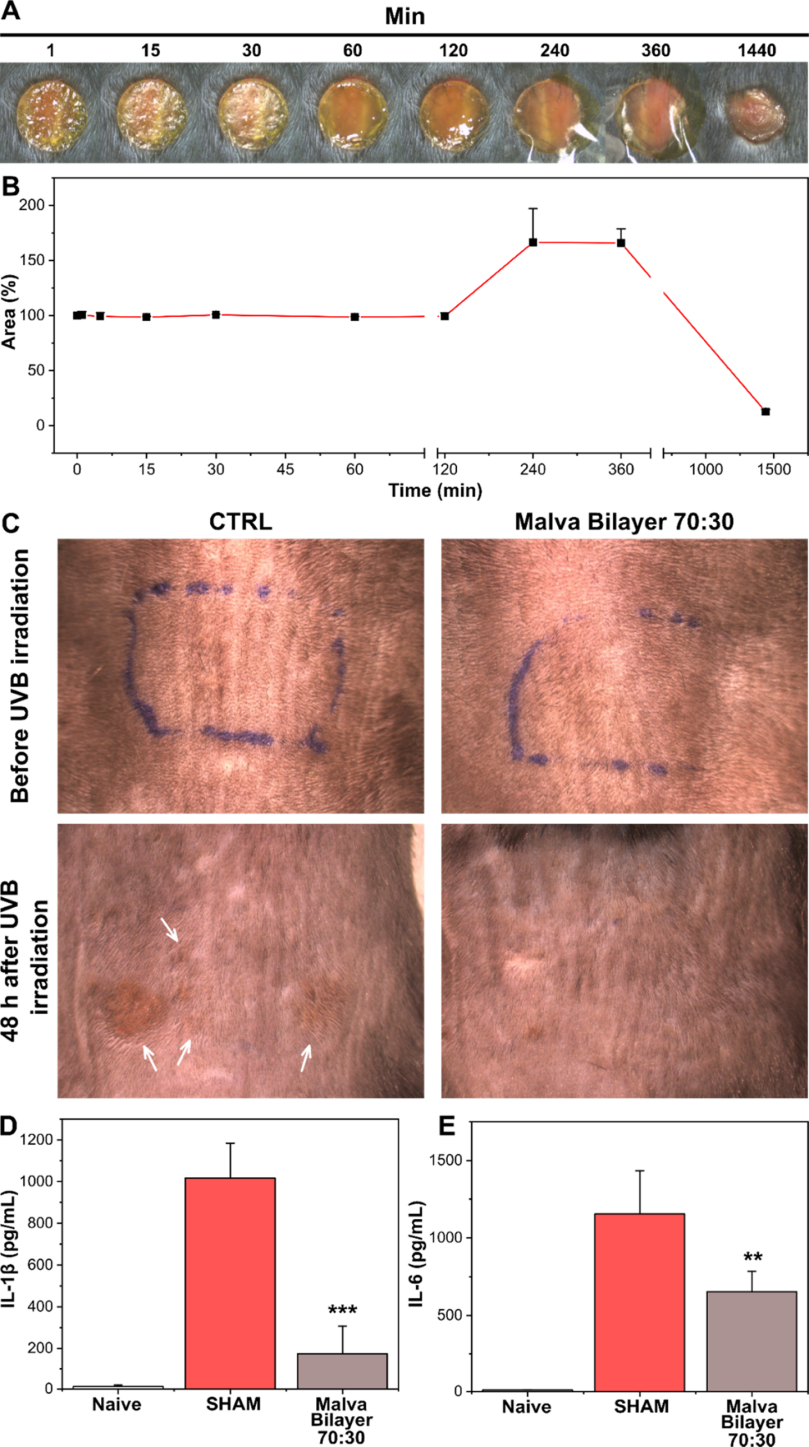
(A) Photographs at different time points of MS bilayer 70:30 applied on a wound. (B) Expression of the resorbed material percentage as a function of time. (C) Photographs of mice dorsal skin before and after UVB exposure of control and MS bilayers. White arrows indicate the damaged skin area. (D, E) Expression of IL-1β and IL-6, respectively, for naïve, Sham, and MS bilayer groups. Results are presented as the average ± SD. **p < 0.01 vs Sham; ***p < 0.001 vs Sham.

After their application on the damaged skin tissue, advanced patches should be resorbed by the skin to not become a physical obstacle to the wound healing process and potentially injure the skin again during their removal. The bioresorption properties of MS bilayer 70:30 were evaluated in an *in vivo* full-thickness excisional skin wound model, and the results are reported in Figure 7A,B. As can be noticed, after the application, the bilayer starts absorbing the present exudate, and after 2 h, its swelling can be observed. In 24 h, 87% of the bilayer is resorbed by the wound. This capacity can allow applying the biomaterial for the second or other times, if necessary.

The anti-inflammatory properties of MS bilayer 70:30 were evaluated in a UVB burn-induced mice model. Photographs of mice skin before and 48 h after the UVB exposition are reported in Figure 7C. After UVB irradiation, MS bilayer 70:30 films were applied to the back of the mice. Large areas of reddish damaged skin were present in the nontreated mice, as can be noticed in Figure 7C. On the contrary, mice treated with the MS bilayer 70:30 did not show any evident redness and alteration of the skin. The levels of the inflammatory mediators IL-1β and IL-6 in the mice’s skin were quantified by ELISA, and the main results are reported in Figure 7D,E. The mice treated with MS bilayers 70:30 showed a statistical reduction of the levels of both inflammatory biomarkers with respect to the nontreated mice. Specifically, decreases of 83 and 43% of the IL-1β and IL-6 levels, respectively, were recorded for the MS bilayers with respect to the controls. These outcomes demonstrated that MS bilayer 70:30 could be a suitable material for the treatment of burns since it is capable of scavenging free radicals produced during such injuries, reducing the local inflammation of the skin tissue and limiting the morphological side effects, such as scars.

## 4 Conclusions

In this work, we presented the introduction of bioactive MS extracts in PVP/alginate-based bilayer films produced by solvent-casting and rod-coating methods. The successful fabrication of the two separate layers was confirmed both by morphological and chemical analyses. The presence of the extract promotes the observed shifts in the polar groups of the polymers and the mechanical properties of the final materials. The MS bilayer materials showed superior adhesive properties compared to a commercial patch and other bilayer materials, strong antioxidant activity, and a quick release of their content, a crucial parameter to immediately block the diffusion of harmful free radicals. In contact with keratinocytes, the bilayers were fully biocompatible. In the *in vitro* wound scratch test, the MS bilayers statistically improved the keratinocytes’ recovery rate compared to the untreated controls and cells treated with the control films, demonstrating the efficacy of the loaded molecules in accelerating cell proliferation. Finally, MS bilayer 70:30, which showed the best performance compared to MS bilayer 50:50, was tested in *in vivo* models. It was fully resorbed in 24 h and showed the capacity to reduce the skin damage and statistically decrease the level of inflammatory mediators IL-1β and IL-6. All of the aforementioned outcomes make the MS extract-loaded bilayer materials suitable for application on the skin for the treatment of burns and wounds characterized by high levels of harmful ROS.

## Supporting Information Available

UV–vis spectrum of the leaf extract in ethanol, LC–MS chromatograms for the flower and leaf extracts, main molecules detected by LC–MS chromatography of the flower and leaf extracts, and release of malvidin and ferulic acid from the bilayers (PDF)
