# Supplementary material for: Self-Adhesive and Antioxidant Poly(vinylpyrrolidone)/Alginate-Based Bilayer Films Loaded with Malva sylvestris Extracts as Potential Skin Dressings
Source: ACS Appl Bio Mater. 2022 May 18;5(6):2880–93. doi: 10.1021/acsabm.2c00254 (PMC9214765; doi:10.1021/acsabm.2c00254)
Supplement: Supplementary file 1 — mt2c00254_si_001.pdf [file mt2c00254_si_001.pdf]

Supporting Information

Self-Adhesive and Antioxidant Polyvinylpyrrolidone/Alginate-based Bilayer Films Loaded with *Malva Sylvestris* Extracts as Potential Skin Dressings

Marco Contardi[‡,a], Amin Mah'd Moh'd Ayyoub[‡,a,b], Maria Summa[c], Despoina Kossyvaki[a,b], Marta Fadda[a,b], Nara Liessi[d], Andrea Armirotti[d], Despina Fragouli[a], Rosalia Bertorelli[c], Athanassia Athanassiou[a,*].*

[a]Smart Materials, Istituto Italiano di Tecnologia, Via Morego 30, 16163, Genova, Italy

[b]Dipartimento di Informatica Bioingegneria, Robotica e Ingegneria dei Sistemi (DIBRIS), Università degli studi di Genova, Via Opera Pia 13, Genova 16145, Italy

[c]Translational Pharmacology, Istituto Italiano di Tecnologia, Via Morego 30, 16163, Genova, Italy

[d]Analytical Chemistry Facility, Istituto Italiano di Tecnologia, Via Morego 30, 16163, Genova, Italy

‡ *These authors contributed equally to this work*

**Corresponding authors:* marco.contardi@iit.it M.C.; athanassia.athanassiou@iit.it A.A.

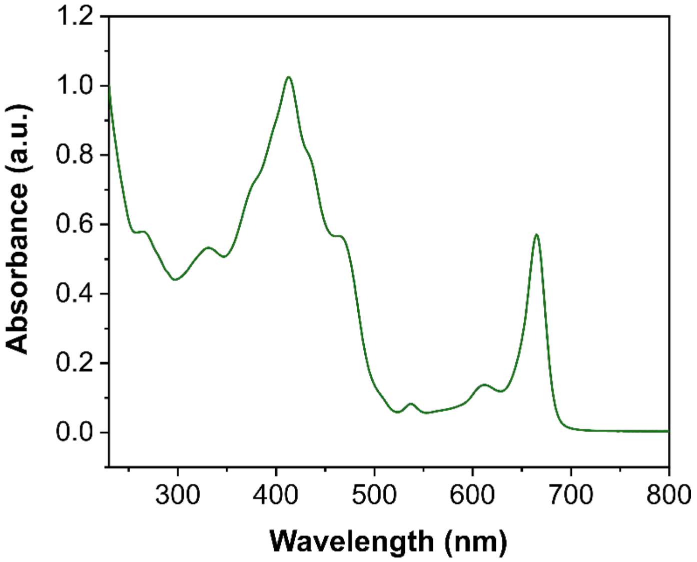

Figure S1. UV-Vis spectrum of leaves extract in ethanol.

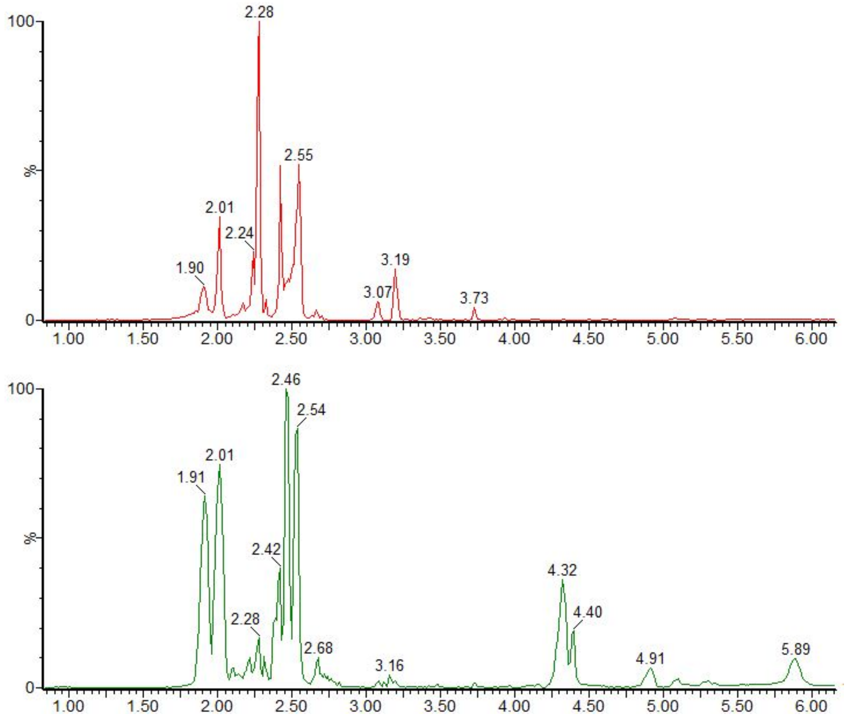

Figure S2. Representative LC-MS chromatograms (positive and negative electrospray modes) for the flower extract.

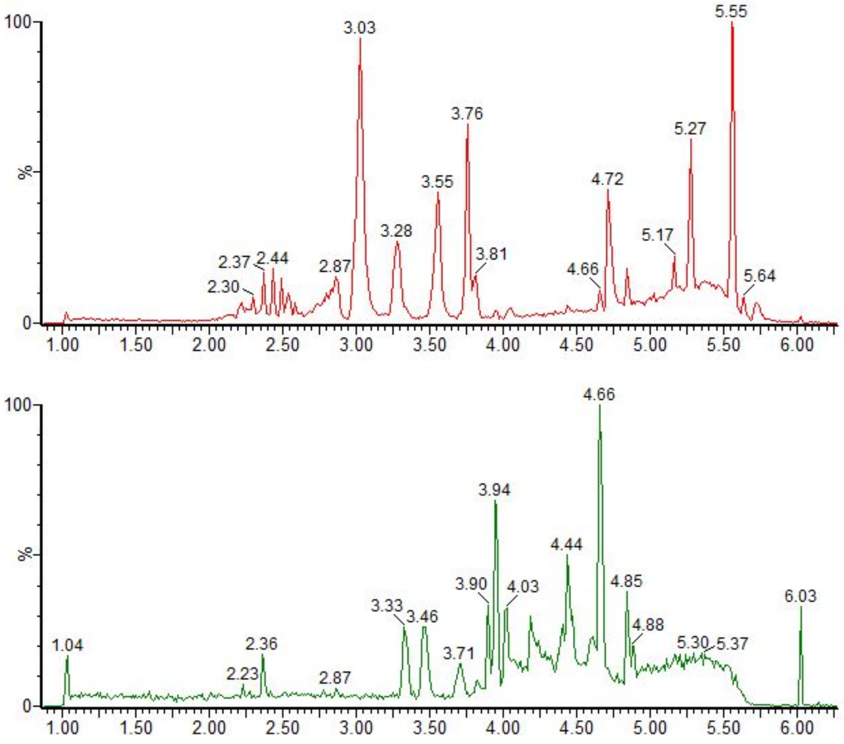

Figure S3. Representative LC-MS chromatograms (positive and negative electrospray modes) for the leaf extract

Polarity	Sample	m/z	MW	Exact Mass	Name
ESI+	Flowers	655,1880	655,5810	655,1869	malvin
ESI+	Flowers	741,1870	741,6270	741,1873	malonylmalvin
ESI+	Flowers	697,1885	696,6110	696,1902	6-[(2S,3R,4S,5S)-4,5-dihydroxy-3-{[(3R,4S,5R)-3,4,5-trihydroxyoxan-2-yl]oxy}oxan-2-yl]-5-hydroxy-2-(4-hydroxyphenyl)-7-{[(2S,3R,4S,5S,6R)-3,4,5-trihydroxy-6-(hydroxymethyl)oxan-2-yl]oxy}chromen-4-one
ESI+	Flowers	792,2189	774,7280	774,2160	(6-{[2-(3,4-dihydroxyphenyl)-3,7-dihydroxy-3,4-dihydro-2H-1-benzopyran-5-yl]oxy}-3,4-dihydroxy-5-{[3-(4-hydroxy-3-methoxyphenyl)prop-2-enoyl]oxy}oxan-2-yl)methyl 3-(4-hydroxyphenyl)prop-2-enoate
ESI+	Flowers	611,1594	610,5210	610,1534	7-{[(2S,4S,5S)-4,5-dihydroxy-6-(hydroxymethyl)-3-{[(2S,4S,5S)-3,4,5-trihydroxy-6-(hydroxymethyl)oxan-2-yl]oxy}oxan-2-yl]oxy}-2-(3,4-dihydroxyphenyl)-5-hydroxychromen-4-one
ESI+	Flowers	465,1150	465,3860	465,1028	5,7-dihydroxy-3-{[(3R,4S,5S,6R)-3,4,5-trihydroxy-6-(hydroxymethyl)oxan-2-yl]oxy}-2-(3,4,5-trihydroxyphenyl)-1λ^4-chromen-1-ylium
ESI+	Flowers	449,1206	448,3800	448,1006	luteolin 7-O-glucoside
ESI+	Flowers	303,0511	303,2450	303,0499	delphinidin
ESI+	Flowers	595,1661	594,5220	594,1585	vicenin 2
ESI+	Flowers	287,0555	286,2390	286,0477	luteolin
ESI+	Flowers	433,1141	432,3810	432,1056	genistin
ESI+	Flowers	331,08	331,2990	331,0812	malvidin

**ESI-**	Flowers	609,1468	610,5210	610,1534	7-{[(2S,4S,5S)-4,5-dihydroxy-6-(hydroxymethyl)-3-{[(2S,4S,5S)-3,4,5-trihydroxy-6-(hydroxymethyl)oxan-2-yl]oxy}oxan-2-yl]oxy}-2-(3,4-dihydroxyphenyl)-5-hydroxychromen-4-one
**ESI-**	Flowers	593,1508	594,5220	594,1585	vicenin 2
**ESI-**	Flowers	431,0981	432,3810	432,1056	genistin
**ESI-**	Flowers	163,03	164,1600	164,0473	hydroxycinnamic acid
**ESI-**	Leaves	193,05	194,186	194,0579088	7-hydroxy-6-methoxy-4a,8a-dihydrochromen-2-one
**ESI-**	Leaves	193,05	194,186	194,0579088	ferulic acid
**ESI-**	Leaves	295,22	296,451	296,2351449	vernolic acid

Table S1. Molecular masses detected in flowers and leaves extract and putative compounds annotated through the METLIN database*.

(*) Smith, C. A.; O'Maille, G.; Want, E. J.; Qin, C.; Trauger, S. A.; Brandon, T. R.; Custodio, D. E.; Abagyan, R.; Siuzdak, G. METLIN: a metabolite mass spectral database. Therapeutic drug monitoring 2005, 27 (6), 747-751.

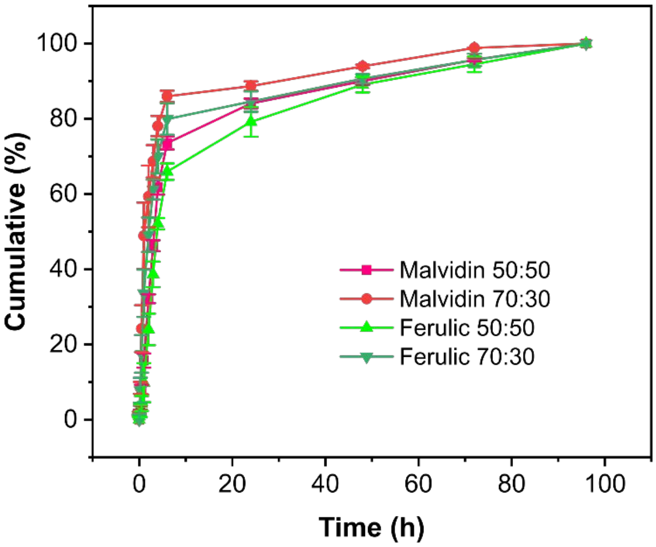

Figure S4. The release profile of Malvidin and Ferulic acid from the Malva bilayer films for 96 hours.
